# Intraarterial embolizations in life-threatening spontaneous retroperitoneal and rectus sheath hemorrhage (SRRSH): a three-center experience

**DOI:** 10.1007/s10140-023-02137-6

**Published:** 2023-04-29

**Authors:** Lena S. Becker, Fabian Stöhr, Volker Maus, Cornelia L.A. Dewald, Bernhard C. Meyer, Frank K. Wacker, Roman Kloeckner, Jan B. Hinrichs

**Affiliations:** 1 Institute of Diagnostic and Interventional Radiology, Hannover Medical School, Carl-Neuberg-Str. 1, 30625 Hannover, Germany; 2 Institute of Diagnostic and Interventional Radiology, Johannes Gutenberg University Mainz, Mainz, Germany; 3 Institute of Radiology, Neuroradiology and Nuclear Medicine, University Hospital Knappschaftskrankenhaus Bochum, Bochum, Germany; 4 Institute for Interventional Radiology, University Clinic Schleswig-Holstein – Campus Lübeck, Lübeck, Germany

**Keywords:** Spontaneuous retroperitoneal hemorrhage, Spontaneous rectus sheath hemorrhage, Transarterial embolization

## Abstract

**Purpose:**

To retrospectively evaluate the technical and clinical success of interventional treatments employed in three University medical centers and to develop work-flow recommendations for intra-arterial embolizations in patients with life-threatening spontaneous retroperitoneal and rectus sheath hemorrhage (SRRSH).

**Materials and methods:**

Retrospective evaluation of all patients with contrast-enhanced CT and digital subtraction angiography (DSA) for SRRSH from 01/2018 to 12/2022, amounted to 91 interventions in 83 patients (45f, 38m) with a mean age of 68.1 ± 13.2 years. Analysis of the amount of bleeding and embolized vessels, choice of embolization material, technical success, and 30-day mortality was performed.

**Results:**

Pre-interventional contrast-enhanced CT demonstrated active contrast extravasation in 79 cases (87%). DSA identified a mean of 1.4 ± 0.88 active bleeds in all but two interventions (98%), consisting of 60 cases with a singular and 39 cases of >1 bleeding artery, which were consecutively embolized. The majority of patients underwent embolization with either n-butyl-2-cyanoacrylate (NBCA; *n*=38), coils (*n*=21), or a combination of embolic agents (*n*=23). While the technical success rate was documented at 97.8%, 25 patients (30%) died within 30 days after the initial procedure, with mortality rates ranging from 25 to 86% between the centers, each following different diagnostic algorithms.

**Conclusion:**

Embolotherapy is a safe therapy option with high technical success rates in patients with life-threatening SRRSH. To maximize clinical success and survival rates, we propose a standardized approach to angiography as well as a low threshold for re-angiography.

## Introduction

Spontaneous retroperitoneal and rectus sheath hemorrhage (SRRSH) represents a potentially fatal condition in the absence of associated trauma, iatrogenic manipulations, or underlying vessel pathology such as a ruptured aneurysm [1–3]. Associated risk factors include the receipt of therapeutic anticoagulation, with incidences expecting to rise as more patients undergo anticoagulation therapy for a variety of diseases [4]. Less common risk factors include hemophilic conditions, hemodialysis, presence of retroperitoneal formations (e.g., tumors), and heart disease or liver dysfunction. Non-specific clinical presentation may delay its diagnosis [5–7] and increase the risk of high-volume blood loss, resulting in high morbidity and mortality rates of up to 30% lethality [8]. Computer tomography (CT) and particularly CT angiography (CTA) play an essential role in (timely) diagnosis and location of active bleeding [9]. Management of SRRSH remains controversial, in the past often favoring conservative approaches including fluid resuscitation, blood transfusion, and coagulation factors [4–11]. While surgical treatments tend to be reserved for hemodynamically unstable patients with abdominal compartment or neurological disorders, there have been a number of studies promoting interventional approaches for bleeding control and overall prognosis improvement [2, 4, 9, 12–14]. Current studies acknowledge active contrast medium extravasation on CT, presence of pain, large hematoma size, and muscular fascia rupture as main indications for diagnostic angiography with consecutive interventional treatment [4, 9, 11, 15]. However, as of yet, no generalized treatment algorithm exists, defining when and how to attempt transarterial embolization (TAE). Furthermore, the documented workflow for interventional treatment in patients with SRRSH in literature demonstrates large variability in oftentimes small-numbered studies. The purpose of our multicentric study was to retrospectively evaluate safety and efficacy of interventional treatments employed in three University medical centers as well as to develop work-flow recommendations for intra-arterial embolizations in patients with life-threatening SRRSH.

## Material and methods

### Study population

**Fig. 1:**
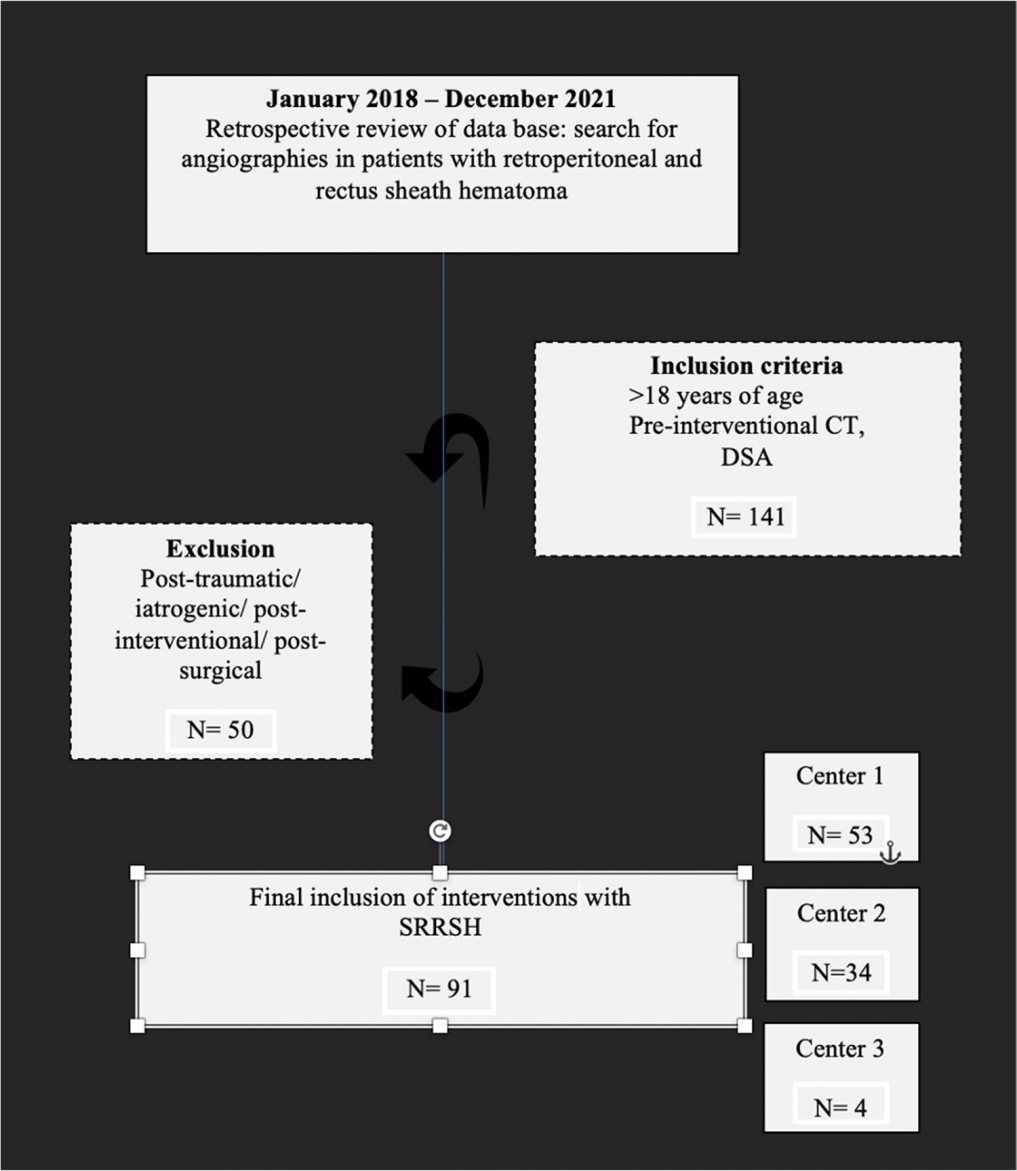
Flowchart providing patient inclusion and exclusion

Three University medical centers ([1] *Hannover Medical School*, [2] *Johannes Gutenberg University Mainz*, [3] *University Hospital Knappschaftskrankenhaus Bochum*) participated in this retrospective study. The study protocol was approved by the individual institutional ethics committees with a waiver of informed consent. From January 2018 through December 2021, all patients with SRRSH were included, who were admitted to one of the three participating tertiary care medical centers and who underwent digital subtraction angiography (DSA) for retroperitoneal and rectus sheath bleeding, indicated by clinical and imaging signs of an active bleeding. To rule out a mechanical or iatrogenic cause of bleeding, we excluded patients with interventional/ surgical procedures ≤ 14 days before DSA (see Fig. 1). Overall, we included 91 interventions in 83 patients (45f, 38m). The mean age was 68.1 ± 13.2 years. All patients were hospitalized, either showing or developing signs of life-threatening hemorrhage during the clinical stay. Initial treatment by emergency medicine specialists included fluid resuscitation, withdrawal of anticoagulant therapy (if possible), and correction treatment for coagulopathy. Retrospective review of the patient’s medical records was performed for a variety of variables, including demographic data, clinical symptoms in accordance with SRRSH (e.g., flank ecchymosis, abdominal mass or pain, generalized weakness), identification of an active bleed in pre-interventional CTA, laboratory findings closest to time of diagnosis (international normalized ratio (INR), activated partial thromboplastin time (APTT), thrombocytes, hemoglobin), documented comorbidities, use of anticoagulants/antiplatelets, history of blood transfusions, duration of hospital stay, intensive care unit (ICU) admission, ICU length of stay, and mortality (for details, see Tables 1, 2 and 3).

**Table 1:** Patient demographics

	Hannover	Mainz	Bochum	**Total**
Screened retroperitoneal bleeding cases	100	34	7	**141**
- Included interventions	53	34	4	**91**
- Excluded interventions (non-spontaneous bleeds)	47	-	3	**50**
Included patients	45	34	4	**83**
- Gender (f;m)	22; 23	20; 14	3; 1	**45; 38**
- Age [years]	67.6 ± 12.4	70.5 ± 12.6	69.8 ± 15.4	**68.1 ± 13.2**
- Anticoagulation (%)	81.4	70.6	50	**75.6**
CT				
- Pre-interventional CT	53	34	4	**91**
- Signs of active bleeding on initial CT o Yes o No	41 12	34 -	4 -	**79** **12**
- Time from CT to angiography [minutes]	137.9 ± 194	94 ± 58	209.5 ± 118	**124.6 ± 155**
Angiographies - Bleeding arteries on DSA				
o *n*= 0	2	-	-	**2**
o *n*= 1	39	17	4	**60**
o *n*= 2	8	7	-	**15**
o *n*= 3	1	7	-	**8**
o *n*= 4	1	3	-	**4**
o Mean	1.19 ± 0.66	1.88 ± 1.04	1	**1.4 ± 0.88**
Transarterial embolizations				
- Intercostal arteries	3	-	-	3
- Epigastric arteries	10	6	-	**16**
- Lumbar arteries	30	28	3	**61**
- Gonadal arteries	3	-	-	**3**
- Iliac arteries	11	-	1	**12**
- Renal arteries	2	-	-	**2**
Embolization material				
- Coils	17	3	1	**21**
- Gelfoam	7	-	-	**7**
- NBCA (+Lipiodol)	9	28	1	**38**
- Coils + Gelfoam	11	-	-	**11**
- Coils + NBCA	3	-	1	**4**
- Coils + Gelfoam + NBCA	2	3	-	**5**
- Vascular plug + stentgraft	1	-	-	**1**
- Embospheres	-	-	1	**1**
- Coils + embospheres	1	-	-	**1**
- None	2	-	-	**2**
Technical success	0.96 ± 0.2	1	1	**0.98**
Repeated angiographies	7	-	-	**7**
Complications	1	-	-	**1**
- Dissection	1	-	-	**1**
ICU stay	0.61 ± 0.49	0.82 ± 0.39	0.82 ± 0.39	**0.72 ± 0.45**
Duration of hospital stay [days]	30.76 ± 28.96	16 ± 12.5	24.75 ± 18.1	**24.85± 25.25**
Clinical success	0.75 ± 0.44	0.79 ± 0.41	0.5 ± 0.58	**0.76 ± 0.43**
- Survival (>30 days) [%]	0.86 ± 0.2	0.56 ± 0.5	0.25 ± 0.5	**0.61 ± 0.49**

The bold entries were supposed to improve comprehensibility

### Computer tomography angiography

All patients received a pre-interventional multiphase CTA in the routinely available CT scanners (SOMATOM Force, SOMATOM Definition AS (Siemens Healthineers, Erlangen, Germany), PHILIPS Brilliance iCT 256 (Philips Healthcare, Eindhoven, The Netherlands)). CTA confirmed the presence of an active bleed in 71/83 patients (85.5%), excluding twelve patients. In these twelve cases, DSA was later performed upon persisting clinical suspicion, with detection of ≥1 bleeding except in one patient. Scan protocols included a tri-phasic protocol in two of the (*center 1*,*2*: *MHH, JGU*), including a contrast-free, an arterial, and a venous scan of the entire abdomen (reconstruction 1 mm section thickness, 120 kV, collimation 128 × 0.625 mm) and a bi-phasic (arterial + venous phase) protocol in one center (center 3: *UKK*; reconstruction 1 mm section thickness, 120 kV, collimation 192×0.6 mm). For CTA, automated bolus tracking in the abdominal aorta for the arterial phase and a delay of 40–60 s for the venous phase was employed. A bolus of 80–120 mL iomeprol (Imeron 350, Bracco Imaging, Milan, Italy) or iopromide (Ultravist, Bayer Healthcare, Berlin, Germany) followed by a 50 mL saline flush was injected at a flow rate of 3–4 mL/s. Coronal and sagittal reformation CT images were analyzed concerning the presence and number of active contrast medium extravasation, pseudoaneurysm formation, and abrupt vessel cut-off, interpreted as signs of active bleeding.

### Angiography and transarterial embolization

**Fig. 2:**
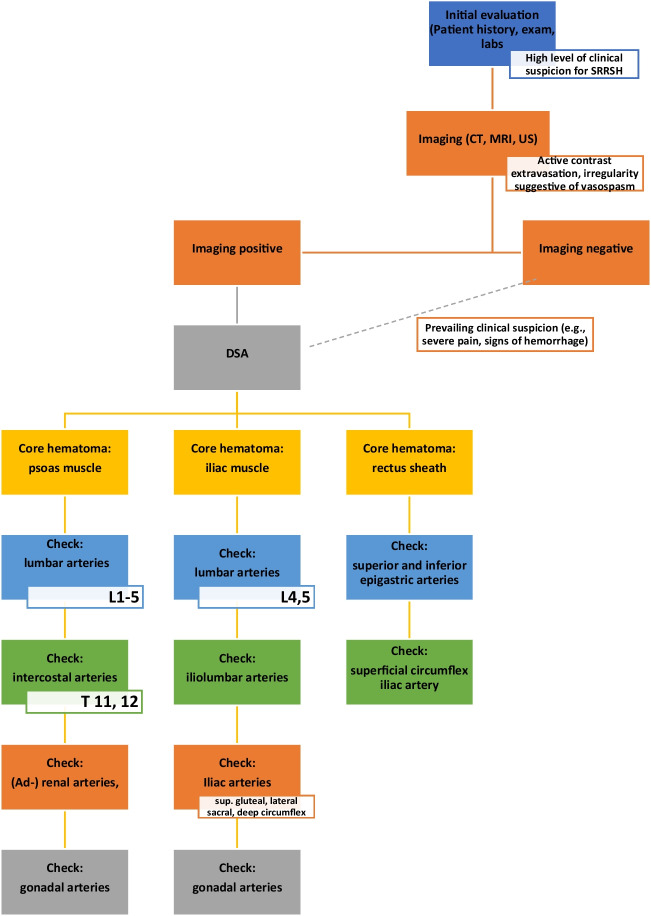
Proposed algorithm for embolic management of SRRSH and schematic visualization of commonly affected blood vessels in retroperitoneal and abdominal wall hematomas: **a** superior epigastric artery, **b** lower intercostal arteries, **c** lumbar arteries, **d** gonadal artery, **e** inferior epigastric artery, **f** iliac arteries, **g** superficial circumflex iliac artery, *adrenal artery

**Fig. 3:**
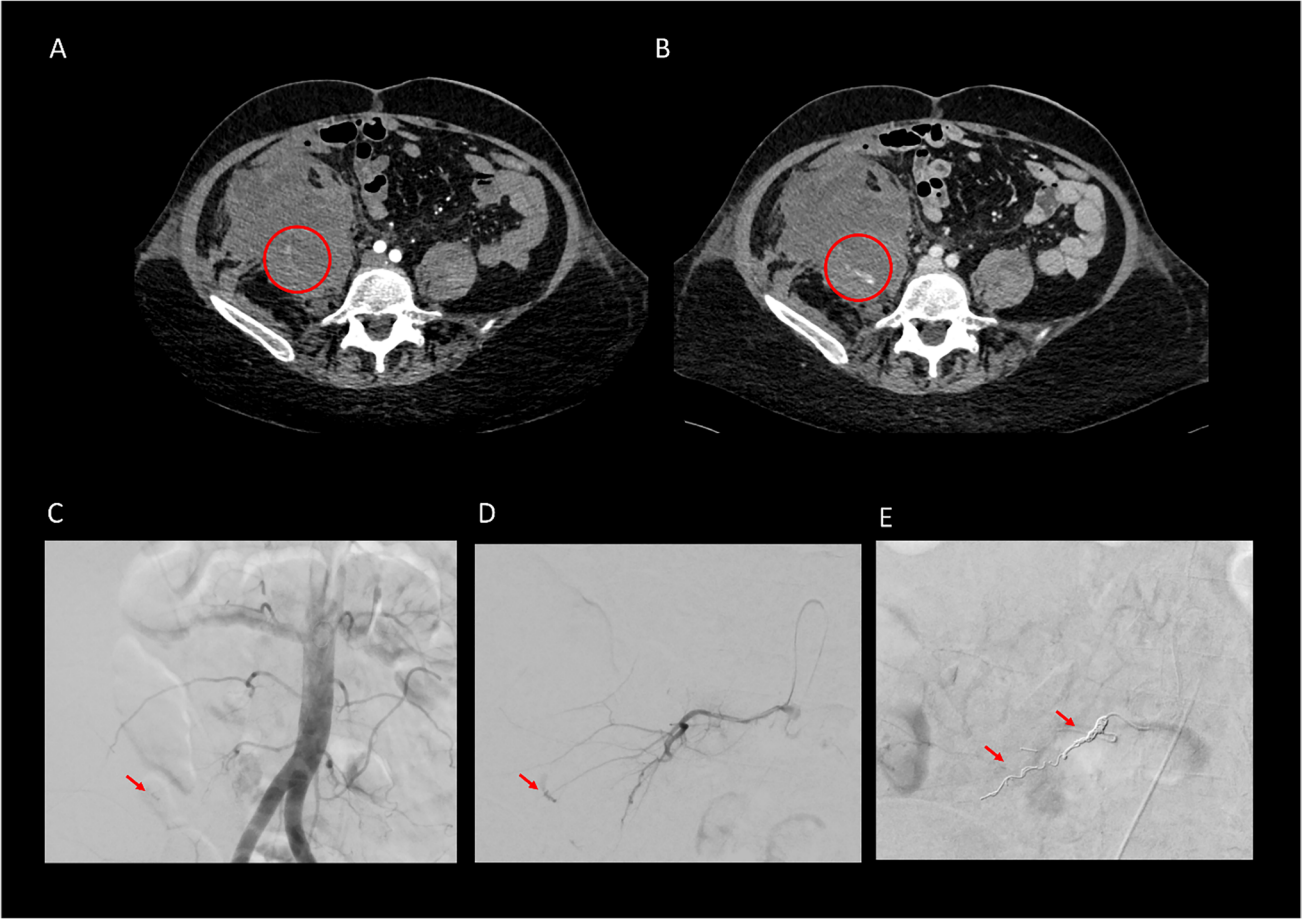
Image example of a patient (52y, f) presenting with hypotension (blood pressure 70/45 mmHg) and abdominal pain. Pre-interventional CT in transversal/coronal/ sagittal planes in the arterial (**A**) and venous phase (**B**) showed an active contrast extravasation in the right iliac muscle as well as a large retroperitoneal hematoma with signs of sedimentation. DSA (**C**) demonstrated an active bleed in iliolumbar branches of the right L4 artery (**C**, **D**), which had been suspected from the CT and which was consecutively embolized with a total of 22 coils (**E**)

**Fig. 4:**
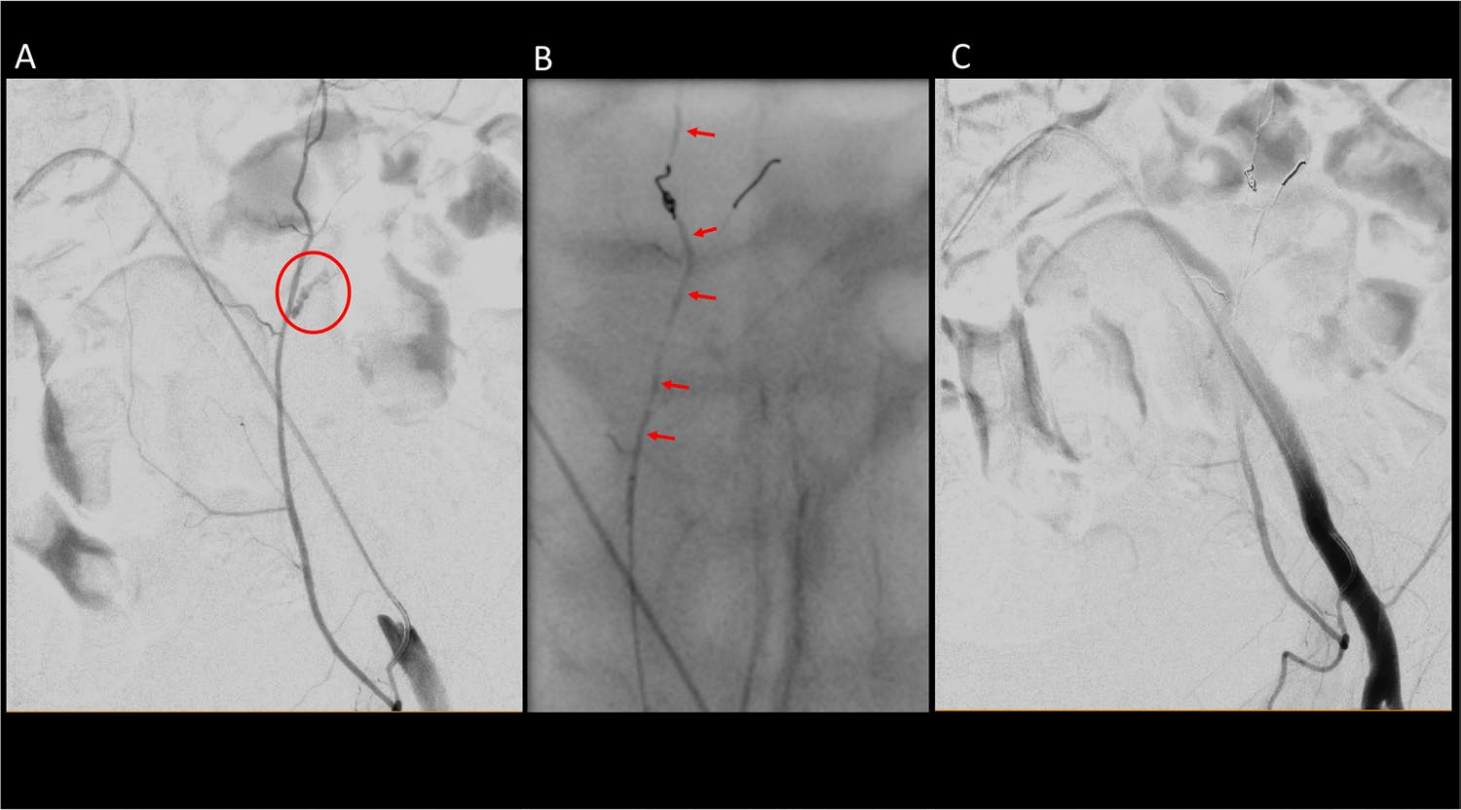
Image example of a patient (78 y, f) presenting with a palpable abdominal mass and abdominal pain. After right-sided femoral access and cross-over catheterization into the left external iliac artery, DSA showed an active contrast extravasation of a branch of the left inferior epigastric artery (**A** red circle). Selective catheterization via microcatheter and coil embolization was accompanied by liquid embolization (NBCA; **B** red arrows), after which the bleeding ceased (**C**)

Angiography was performed either after diagnosis of active bleeding in CTA or upon prevailing clinical suspicion of SRRSH (e.g., severe abdominal pain or continued hemoglobin loss), after discussion in an interdisciplinary taskforce including surgery and internal medicine. Axiom-Artis angiography units (Siemens Healthineers, Erlangen, Germany) and PHILIPS Azurion 7 (Philips Healthcare, Eindhoven, The Netherlands) were used for DSA and transarterial embolization. Four- or 5-French (F) sheaths were introduced via the common, preferably right-sided, femoral artery. In case of unknown or unconfirmed sources of bleeding, arteriograms of the first-order aortic branches, the iliac and femoral arteries were performed, followed by selective arteriograms of the involved arteries suspected on CTA via a suitable microcatheter. Independent of existing, presupposed bleeding sites or of their successful embolization, a standardized search technique was implemented at the site of center 1 (*MHH*), gauging commonly involved vessels in the vascular territory of the core hematoma. In case of a hematoma primarily involving the psoas muscle, lower intercostals (Th 11-12), lumbar (L1-L3), (ad-) renal, and gonadal arteries were interrogated for signs of active contrast extravasation (see Fig. 2). The algorithm sequence in case of an iliac hematoma consisted of a special focus on the lower lumbar arteries (L4–L5), the iliac, and (ad)renal as well as the gonadal arteries. In abdominal wall hematomas, interrogation of the superior and inferior epigastric arteries would be extended by gauging the iliacs and superficial circumflex arteries. Upon the aforementioned signs of active bleeding (e.g., active contrast extravasation, pseudoaneurysm formation), a suitable microcatheter was inserted and preferably advanced distally to the bleeding, in order to embolize the respective vessels in a “front-/backdoor” technique. N-butyl cyanoacrylate (NBCA) and iodised oil (Lipiodol Ultra Fluid, Guerbet, Villepinte, France), mixed at ratios from 1:2 to 1:6, with or without coils, were the most often used embolic materials of choice. Singular use of coils, gel foam, or of use in combination was also documented. In few cases, additional or even singular use of gelatin sponge particles were performed at the interventionalist’s discretion, dependent upon anatomy and catheter position in the target vessel. A list of the embolized arteries and the embolization material can be found in Table 1 and 2. After embolization, the vascular areas above and below the embolized vessels were catheterized selectively in center 1 (*MHH*), thereby excluding collateral flow to (re-) entertain the bleeding (for patient examples: see Figs. 3 and 4). In a single patient, no embolization was performed, as an initially detected bleed on CTA could not be reproduced neither on initial nor on repeated DSA the following day. Major and minor adverse events were documented in accordance with the Society of Interventional Radiology [16] and the CIRSE classification [17] system.

**Table 2:** Subgroup analysis of expired patients

	Expired	Survivors	Influence on survival
Gender	25 (14f; 11m)	58 (32f; 26m)	*p*= 0.543
Age [years]	70.25 ± 13.32	67.47 ± 13.79	*p*= 0.564
Pre-interventional CT			
- Signs of active bleeding	0.91 ± 0.29	0.84 ±0.36	
- Time from CT to angiography [min-max; minutes]	111.25±75.16 [36; 300]	125.92±175.75 [8 ;1074]	*p*= 0.895
Angiographies			
- Bleeding arteries on angiography [min-max]	1.78 ± 1.09 [1; 4]	1.29 ± 0.79 [1; 4]	*p*= 0.017
Transarterial embolizations			
- Embolized arteries	0.95 ± 0.21	0.92 ± 0.27	*p*= 0.04
- Technical success	1	7	
- Repeated angiographies	1	-	
- Complications	1	-	
o Dissection	1	-	
ICU stay	1 (100%)	0.62 ± 0.49	
Duration of hospital stay [days]	19 ± 14.69	27.9 ± 27.52	*p*=0.491
Clinical success			
- Survival (>30 days) [%]	0	100	
- Cause of death			
o Hemorrhagic shock, multiorgan failure	22	-	
o Mesenteric ischemia	1	-	
o Septic shock	2	-	
Laboratory values			
- APTT_pre	46.44 ± 11.6	43.92 ± 20.89	*p*= 0.953
- APTT_post INR_pre	42.2 ± 16.27 2.0 ± 1.26	38.8 ± 16.26 1.56 ± 1.06	*p*= 0.378 *p*= 0.431
- INR_post	1.43 ± 0.45	1.28 ± 0.37	*p*= 0.892

### Laboratory values, follow-up

Laboratory values including hemoglobin, thrombocytes, acute thromboplastin time (APTT), and the international normalized ratio (INR) before and within 24 h of transarterial embolization were documented and compared (see Table 3). Technical success was defined as complete occlusion of the target vessel(s), while clinical success was demonstrated by a stabilization of vital parameters (e.g., rise in blood pressure, decrease in heart rate, hypovolemic shock reversal), indicating permanent occlusion of the bleeding site as well as patient survival > 30 days. A re-bleeding was either proven on repeated imaging or supposed in the absence of stabilization of vital parameters. Receipt of erythrocytes and/or thrombocytes, pre-interventional anticoagulation therapy, and the duration of the entire hospital as well as the intensive care unit stay were documented.

**Table 3:** Comparison of pre- and post-interventional laboratory values

	HB_pre	HB_post	Transfusion	Thombocytes_pre	Thrombocytes_post	APTT_pre	APTT_post	INR_pre	INR_post
MHH	8.7± 2.16	9.3± 4.09	Yes	210.6 ± 215.74	178.88 ±119.71	43.84 ±19.59	42.52 ± 17.06	1.54 ± 1.09	1.34 ± 0.37
JGU	7.4± 1.82	8.99± 0.89	Yes	195.5 ±123.39	157.56 ± 83.88	46.14 ± 18.18	36.19 ± 13.21	1.88 ± 1.21	1.29 ± 0.35
UKK	9.0± 1.35	8.25 ± 1.01	Yes	145.5 ± 92.05	114.25 ±74.19	46.63 ± 13.93	54.88 ± 20.89	1.58 ± 0.52	1.74 ± 0.59

### Statistical methods

The statistical analysis was performed using IBM SPSS Statistics software (Version 27, IBM, Armonk, NY, USA). Numerical values were reported as means with standard deviations (sd), while categorical variables were reported as frequencies and percentages. Univariate analyses were performed using the two-sided Student’s *t* test or the Mann–Whitney *U* test for numerical variables as appropriate (e.g., laboratory values pre- and post-intervention, time to angiography). The Pearson’s chi-squared test or the Fisher’s exact test was used for categorical data, including potential correlations between survival and embolization material, gender, and the individual center. Statistical significance was defined as a *p*-value < 0.05.

## Results

### Study population

Ninety-one interventions in 83 patients (45f; 38m) with a mean age of 68.1± 13.2 years (range: 24-88 years) were included in this study. The majority of the patients (75.6%) received anticoagulants and/or antiplatelet medication at the time of SRRSH occurrence, mainly for presence of atrial fibrillation, preceding valve operations or dialysis. Symptoms upon hospitalization mainly comprised of abdominal/inguinal pain or signs of hemorrhagic shock. Analysis of laboratory values demonstrated an increase of post-interventional hemoglobin from a mean of 8.19 ± 1.33 to 9.14 ± 2.99, post-interventional APTT and INR decreased to 40.31± 16.09 and 1.34 ± 0.38 respectively (pre-interventional APTT: 45 ± 18.55, pre-interventional INR: 1.7 ± 1.13; details see Table 3). Neither analysis of pre- to post-interventional APTT norINR values demonstrated significant correlations with survival (*p*>0.05).

### Imaging (CTA, DSA)

**Fig. 5:**
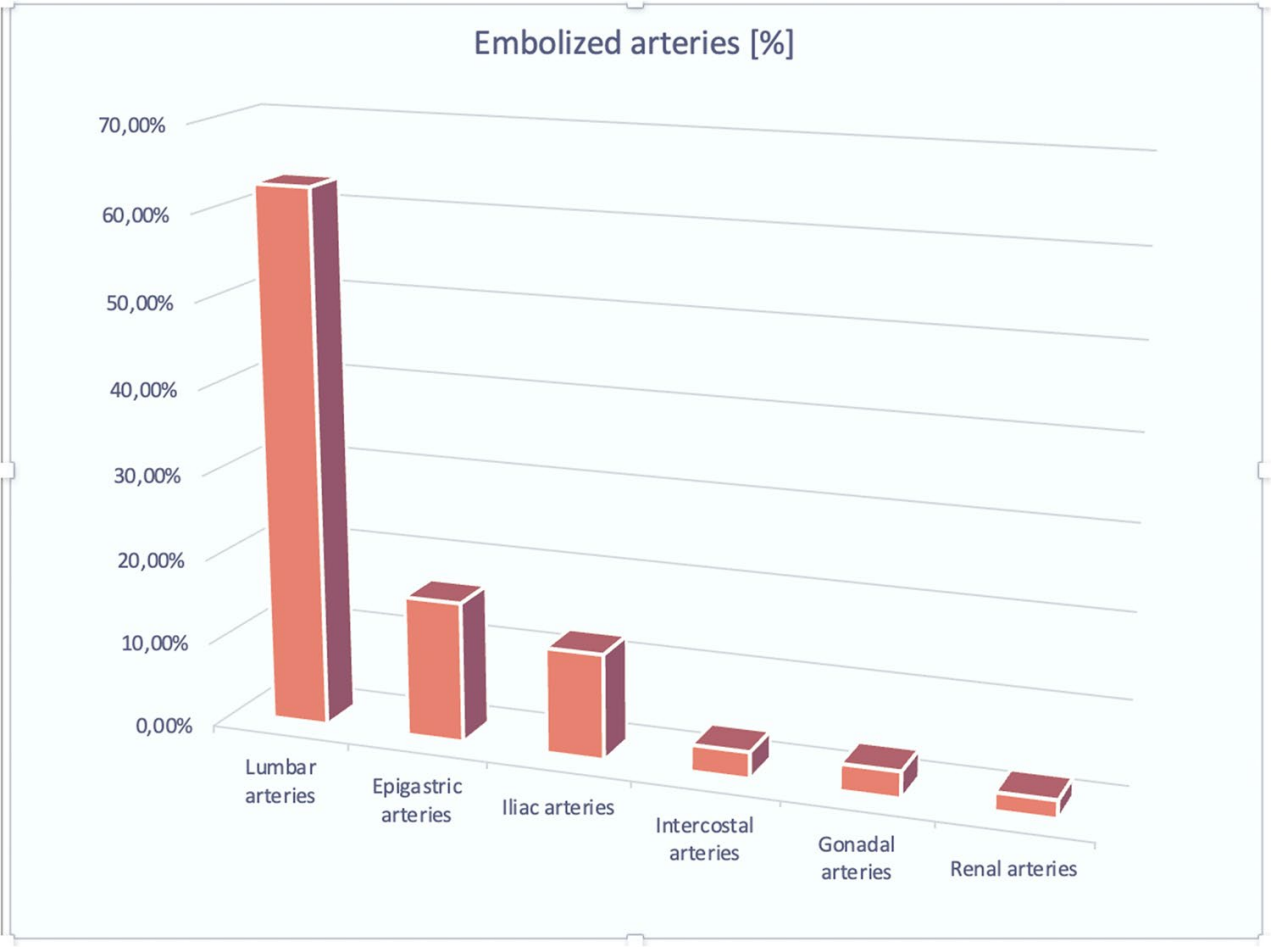
Column chart of the embolized arteries

All patients received a pre-interventional CTA which demonstrated an active bleed in 71 of 83 cases (85.5%). Twelve (13.2%) cases received angiography for persisting clinical suspicion of hemorrhage. DSA demonstrated active bleeds in eleven of these twelve cases (98.8%), exempting one patient, in whom the initial, CTA-based bleeding suspicion, could not be substantiated. In all but this aforementioned patient, transarterial embolizations of a mean of 1.4 actively bleeding arteries (range: 1–4 active bleeding sites) from lumbar arteries (*n*= 53), iliolumbar arteries (*n*=12), epigastric arteries (*n*=16), gonadal (*n*=3), intercostal arteries of the 11th and 12th intercostal space (*n*=3), and renal arteries (*n*=2) were performed (see Fig. 5). Embolotherapy included either the singular use of NBCA and iodised oil (*n*=38), the singular use of coils (*n*=21), the singular use of gel foam (*n*=7) or a combination of NBCA and coils (*n*=11), and gel foam and coils (*n*=9). The technical success rate of the performed embolizations amounted to 98%; the two technically unsuccessful cases received singular coil (*n*=1) and gel foam (*n*=1) embolotherapy, respectively. Clinical success, defined by stabilization of vital parameters (e.g., a rise in blood pressure, decrease in heart rate, hypovolemic shock reversal) after a permanent occlusion of the bleeding site as well as patient survival > 30 days, amounted to 76.7%± 0.43. Four patients received repeated angiography 1 day after initial DSA: *n*=3 due to recurrent bleeding — though with embolization of different vessels than during the initial DSA — and *n*=1 patient due to persisting clinical suspicion without proof of active bleeding.

### Follow-up

Mean duration of hospitalization lays at 24.85± 25.25 days, which included stays in the ICU for 72.3% (*n*=60) of patients. Twenty-five patients did not survive within the follow-up period of 30 days, amounting to a mean lethality rate of 27.5%. Causes of death included hemorrhagic shock with consecutive multiorgan failure (*n*=22), septic shock (*n*=2), and a severe case of mesenteric ischemia (*n*=1). Subgroup analysis of the deceased patients (14f, 11m) demonstrated a higher, yet insignificant mean age at time of death at 70.25 ± 13.32 years compared to the surviving patients at 67.47 ± 13.79 years (*p*= 0.56), higher rates of confirmed signs of active bleeding (91.3% vs. 84.6%; *p*= 0.017), and slightly shorter delays from CTA to angiography of 111.25 ± 75.12 min vs. 125.93 ± 275.75 min (*p*= 0.9). However, no significant correlation between time to angiography and survival could be found (*p*= 0.96). The number of bleeding (and consecutively embolized) vessels lays at 1.78 ± 1.09 in the deceased group and at 1.29 ± 0.79 in the survivors’ group and demonstrated a significant impact on survival (*p*= 0.017, *p*=0.004). Technical success rates were slightly lower in the group of the deceased patients at 95.5 ± 21.3% compared to 98.4 ± 12.5% in the cohort of survivors (details: see Table 2).

## Discussion

Despite being recognized as a potentially life-threatening condition, relatively little has been reported about retroperitoneal and rectus sheath hemorrhage since its first description approximately 60 years ago [18]. Next to associations with anticoagulant therapy, hemodialysis, trauma, surgery, and/or underlying vascular pathologies, spontaneous occurrences without precipitating factors have been reported and are expected to increase in an aging population receiving anticoagulants for various indications, including atrial fibrillation, acute coronary syndrome, or deep vein thrombosis [2, 4, 18, 19]*.* A well-known complication of anticoagulation therapy, acute hemorrhage has been described with incidences of 0.6 to 6.6% in the retroperitoneum and adominal wall [2, 3]. The majority of the patients in our multicentric study were anticoagulated at the time of the SRRSH, which is in line with aforementioned research [2, 4]. Sensitive diagnostic tools are essential in light of unspecific clinical findings or an insidious onset, potentially delaying a timely diagnosis and increasing the risk of shock, compartment syndrome, or even exsanguination.

Both CTA (86%) and DSA (96%) demonstrated a high prevalence of active bleeding in this study, reaffirming them as sensitive tools when searching for active bleeds in the retroperitoneal space and/or the abdominal wall. However, in twelve cases in this study, initial CTA did not demonstrate the suspected bleeding and would have been missed in case of DSA omittance. This highlights the importance of retaining a high suspicion of active bleeding and a low threshold for acquisition of diagnostic angiography even in seemingly less acute cases [20]. A similar concept of increased vigilance may be applied to the search of bleeding sites during angiography, as SRRSH has been suggested to be a manifestation of a more diffuse process with the potential of multiple bleeding sites and a multifaceted treatment due to complex vascularization with multiple anastomoses [18]. Groups such as Contrella et al. have consecutively described performing arteriograms of selected contralateral arteries, if the preprocedural image raised concern for hemorrhage from this vessel [11] and Farrelly et al. suggested the importance of interrogating all vessels (potentially) supplying a region occupied by the hematoma on CT [14]. However, no previous group performed a systematic search of these vessels or has shown the benefit of such an approach. In dependence on the region occupied by the hematoma, different pathways of the proposed algorithm were followed in center 1 (see Fig. 2). By expanding the search perimeter for bleeding sites and performing a structured, sequential search, center 1 (*MHH*) discovered and embolized a larger and more diverse amount of bleeding arteries, significantly impacting survival. This underlines the importance of following a standardized approach and checking even unexpected involved vessels, e.g., gonadal arteries. Found to be the source of bleeding in three cases in this study, they have (to our knowledge) not been featured prominently in previous literature.

Therapy rests upon different pillars, ranging from conservative management, over open surgery to transarterial embolotherapy methods, which have demonstrated a good safety profile and high technical success rates [2, 20]. Defined as a cessation of extravasation and occluded or stagnant flow in the embolized vessel, high technical success rates, however, do not necessarily correspond with survival of the patients, possibly due to multiple organ dysfunction syndrome (MODS) and/or incomplete target vessel occlusion. With reported 30-day mortality rates of up to 30% [2, 8, 21, 22], SRRSH remains a hazardous and potentially life-threatening disease even >60 years after its initial description. The use of various embolic materials such as gelfoam, microparticles, microcoils, and NBCA in heterogeneous combinations has been described [21–23]. However, no direct comparison and no recommendation consensus concerning the choice of embolic agents or the generalized workflow exist as of yet. Up until recently, widespread use of coils was reported, while the latest studies demonstrate a preference for liquid embolic agents with or without additional coil placement [2]. Independent of the coagulation status of patients, which may appear deranged especially under anticoagulation, NBCA allows for swift and dependable vessel occlusion [24]. A preference for embolization with cyanoacrylates is mirrored in this study, though potential complications such as tissue ischemia, systemic or local reactions, and catheter adhesion to blood vessel walls or glue fragmentation with inadvertent embolization [25–27] have also created an aftertaste of inherent unpredictability, curtailing NBCA’s popularity and preventing its ubiquitous use.

While of limited comparability due to differing inclusion numbers and a high number of potential confounders, lethality rates within the centers differed, ranging from 25 to 86.5%. For center 1 (*MHH*), the overall 30-day mortality rate of patients receiving either singular NBCA or a combination of NBCA and coils (8.5%) was lower than that of deceased patients receiving singular coil or gel foam embolotherapy (11.9%), and thus lower than previously reported 30-day mortality rates of 10–30% [2, 5, 8, 21–23]. In addition, the two patients receiving up to three re-angiographies with recurrent bleedings in the same vessel territory, had received embolization only by gelfoam, potentially indicating that the sole use of gelfoam embolization materials may not be sufficient in some cases of SRRSH. With recurring bleeding in the same vascular territory, an inefficient embolization must be assumed, while new sources of bleeding after initially successful embolization may very well be associated with disrupted vessels by an expanding retroperitoneal or rectus sheath hematoma [10]. While most certainly being influenced by various confounders, possible explanations for the different lethality rates between the centers may partly be found in the different procedure workflows: as mentioned above, a standardized search technique was implemented at the site of center 1 that involved the survey of commonly involved vessels in the literature, preferably within but also beyond the presumed vascular territory of the core area of the hematoma. This seems plausible as a high number of collateral vessels in the retroperitoneum and the abdominal wall is known, which can complicate embolizations in “front-door/back-door” technique [2]. Neither at center 2 (*JGU*) nor at center 3 (*UKK*) were repeat angiographies reported, while at center 1, this pertained to four patients, hinting at possible advantages to a lower threshold for re-angiography upon symptom persistence.

With the exception of one inadvertent focal dissection without flow limitation, no minor or major, procedure-related complications occurred in our study after NBCA use, indicating an adequately safe and efficient use of embolization. However, this was a retrospective study with heterogeneous patient cohorts and therapy approaches, decreasing comparability between the centers.

In conclusion, larger, prospective studies are needed to address the existing knowledge gap and to study the optimal clinical management strategies for patients with SRRSH. However, its high fatality rates can be potentially decreased by a methodical, standardized approach of performing selective catheterization of arterial feeders in the core of the hematoma as well as a lower the threshold for re-angiography.

## Abbreviations

**CT(A)**: Computer tomography (angiography)
**DSA**: Digital subtraction angiography
**F**: French
ICU: Intensive care unit
**JGU**: Johannes Gutenberg University Mainz
**MHH**: Hannover Medical School
**NBCA**: n-Butyl cyanoacrylate
SRRSH: Spontaneous retroperitoneal and rectus sheath hemorrhages
Sd: Standard deviation
TAE: Transarterial embolization
**UKK**: University Hospital Knappschaftskrankenhaus Bochum
